# An Integrated Approach to Identify New Anti-Filarial Leads to Treat River Blindness, a Neglected Tropical Disease

**DOI:** 10.3390/pathogens10010071

**Published:** 2021-01-14

**Authors:** Rahul Tyagi, Christina A. Bulman, Fidelis Cho-Ngwa, Chelsea Fischer, Chris Marcellino, Michelle R. Arkin, James H. McKerrow, Case W. McNamara, Matthew Mahoney, Nancy Tricoche, Shabnam Jawahar, James W. Janetka, Sara Lustigman, Judy Sakanari, Makedonka Mitreva

**Affiliations:** 1 Division of Infectious Diseases, Department of Medicine, Washington University School of Medicine, 4523 Clayton Ave., St. Louis, MO 63110, USA; rtyagi@wustl.edu; 2 Department of Pharmaceutical Chemistry, University of California San Francisco, 1700 4th Street, San Francisco, CA 94158, USA; christina.bulman@ucsf.edu (C.A.B.); chelsea.fischer811@gmail.com (C.F.); Michelle.Arkin@ucsf.edu (M.R.A.); 3 ANDI Centre of Excellence for Onchocerciasis Drug Research, Biotechnology Unit, Faculty of Science, University of Buea, Buea CM-00237, Cameroon; fidelis.cho@ubuea.cm; 4 Division of Neurocritical Care and Hospital Neurology, Department of Neurology, Mayo Clinic, 200 First Street SW, Rochester, MN 55905, USA; marcellino.christopher@mayo.edu; 5 Skaggs School of Pharmacy and Pharmaceutical Sciences, University of California, San Diego, CA 92093, USA; jmckerrow@ucsd.edu; 6 Calibr, a Division of The Scripps Research Institute, 11119 Torrey Pines Road, La Jolla, CA 92037, USA; cmcnamara@scripps.edu; 7 Department of Biochemistry and Molecular Biophysics, Washington University School of Medicine, 660 S. Euclid Ave., St. Louis, MO 63110, USA; matthew.mahoney@wustl.edu (M.M.); janetkaj@wustl.edu (J.W.J.); 8 Lindsley F. Kimball Research Institute, New York City, NY 10065, USA; NTricoche@nybc.org (N.T.); shabnam.mob@gmail.com (S.J.); SLustigman@nybc.org (S.L.); 9 McDonnell Genome Institute, Washington University School of Medicine, 4444 Forest Park Ave., St. Louis, MO 63108, USA

**Keywords:** parasitic nematodes, filarial nematodes, whole worm assay, in vitro, target class repurposing, anthelmintics, macrofilaricides

## Abstract

Filarial worms cause multiple debilitating diseases in millions of people worldwide, including river blindness. Currently available drugs reduce transmission by killing larvae (microfilariae), but there are no effective cures targeting the adult parasites (macrofilaricides) which survive and reproduce in the host for very long periods. To identify effective macrofilaricides, we carried out phenotypic screening of a library of 2121 approved drugs for clinical use against adult *Brugia pahangi* and prioritized the hits for further studies by integrating those results with a computational prioritization of drugs and associated targets. This resulted in the identification of 18 hits with anti-macrofilaricidal activity, of which two classes, azoles and aspartic protease inhibitors, were further expanded upon. Follow up screening against *Onchocerca* spp. (adult *Onchocerca ochengi* and pre-adult *O. volvulus*) confirmed activity for 13 drugs (the majority having IC_50_ < 10 μM), and a counter screen of a subset against *L. loa* microfilariae showed the potential to identify selective drugs that prevent adverse events when co-infected individuals are treated. Stage specific activity was also observed. Many of these drugs are amenable to structural optimization, and also have known canonical targets, making them promising candidates for further optimization that can lead to identifying and characterizing novel anti-macrofilarial drugs.

## 1. Introduction

River blindness (onchocerciasis) and lymphatic filariasis (LF) are two major neglected tropical diseases (NTD) caused by parasitic nematodes that, together, affect millions of people worldwide in mostly poor, developing countries [1]. Approximately 20 million people are infected with *Onchocerca volvulus*; 14.6 million of the infected people have skin disease, 1.2 million people are visually impaired, and 270,000 are blind by river blindness, a chronic disease caused by the first larval stage, microfilariae (mf). Mf are released from female worms residing in subcutaneous tissues and migrate throughout the skin causing severe itchiness, as well as inflammatory responses in the skin or eyes. When the inflammatory responses occurs in the eye they may eventually lead to impaired vision and ultimately to blindness. LF (elephantiasis) is caused by damage to lymphatic tissues by adult *Wuchereria bancrofti*, *Brugia malayi* and *B. timori* worms, and is characterized by pain and severe lymphedema, often involving the extremities leading to great economic losses as well as social stigma [1].

To date, there are no vaccines to prevent these diseases, and no drugs that directly kill the adult stages (macrofilaricidal drugs) [2,3,4] and can be used in mass drug administration (MDA). A promising triple drug regimen for LF that has some macrofilaricidal effects is currently being evaluated against onchocerciasis [5,6,7]. International control programs attempt to interrupt transmission of infection with annual or biannual MDA using microfilaricidal drugs (ivermectin since 1989 and more recently also moxidectin [8] for onchocerciasis; albendazole and ivermectin or diethylcarbamazine for LF) that kill mf over the lifetime of the adult worms (10–14 years for *O. volvulus*, 6–8 years for *Wuchereria* and *Brugia* spp.) [2,3,4,9,10,11,12,13,14,15,16,17]. These drugs target different critical processes in the mf or make them more susceptible to immune system, e.g., targeting microtubule polymerization (albendazole), glutamate-gated chloride channels and other transporters (ivermectin and moxidectin), and sensitizing microfilariae to phagocytosis by host immune cells (diethylcarbamazine).

Given the longevity and high fecundity of these worms and the current lack of macrofilaricidal drugs, it is unlikely that the WHO goal of eliminating LF and onchocerciasis by 2030 [18,19] will be met using only microfilaricidal drugs [11,20,21]. According to the 2013 Global Burden of Disease Study, only a 31% reduction in onchocerciasis was achieved since MDA with ivermectin began in the 1990s [22]. Indeed, the African Programme for Onchocerciasis Control estimated that elimination of onchocerciasis would require some 1.15 billion treatments with ivermectin (IVM), with MDA efforts continuing until 2045 [11,23,24,25]. It is believed that moxidectin, a potent microfilaricide approved by the FDA to treat human onchocerciasis in 2018 [26], could substantially reduce the time it will take to eliminate onchocerciasis [8], as moxidectin has a superior clinical performance compared to ivermectin [27,28]. This would be especially helpful in places where resource limitation prevents a biannual ivermectin strategy, since biannual ivermectin and annual moxidectin treatment has been shown to achieve similar reductions in program duration [29].

Moreover, MDA with IVM is also confounded in Africa by the fact that it cannot be distributed in areas co-endemic for *Loa loa* (another filarial nematode), due to the risk of severe adverse events, especially toxic encephalopathy when infected individuals have high loads of *L. loa* mf [23,24,30,31]. Presently, treatment in these areas requires a test-and-treat approach, which is more resource-intensive and may result in incomplete treatment for *O. volvulus* [32]. The prohibitive use of IVM for 12 million people, in 11 *Loa*-affected central African countries, impedes elimination efforts and creates a reservoir of *Onchocerca* infections which can re-infect neighboring communities [30]. In addition, LF and onchocerciasis elimination programs in sub-Saharan Africa do not implement MDA in hypoendemic areas (low prevalence of infections), also leading to concerns of putative spreading of reinfections [11] in areas that might have controlled transmission. Furthermore, the potential emergence of IVM-resistant *O. volvulus* limits the long-term effectiveness of present MDA with IVM [4,33,34,35,36], and in time may undermine gains achieved by the MDA programs. The restrictions on MDA in children adds to the complexity of elimination efforts. IVM is not administered to children under 5 years of age, and the only proven indirect macrofilaricidal drug, doxycycline cannot be given to children under 9 years because of drug contraindications. Children thus remain vulnerable and serve as reservoirs of transmission [31]. Moreover, doxycycline requires long treatment periods of 4–6 weeks, which is not feasible for MDA programs [37].

Thus, it is still critical to identify and develop novel, effective and safe macrofilaricidal drugs for use in integrated anti-filarial MDA programs. A few approaches have been studied, including targeting respiratory enzymes and using antibiotics (e.g., doxycycline) effective against *Wolbachia* – an endosymbiont essential to survival of many filarial worms [38]. Ultimately, macrofilaricidal drugs will also have the potential to shorten the time to successfully eliminate onchocerciasis. Unfortunately, there is no animal model to facilitate harvesting of adult *O. volvulus* worms, and even the pre-adult developmental stage (OvL5) which can be used in motility and viability in vitro assays [39] is prohibitively expensive for large scale screenings. Therefore, in this study, we decided on a new stepwise screening approach, with initial large-scale screenings against adult *B. pahangi* worms, followed by secondary screenings of prioritized hits with adult *O. ochengi* worms (a closely related and readily accessible surrogate of *O. volvulus* and a clinical model of human onchocerciasis), and then with pre-adult L5 worms of *O. volvulus*.

In order to facilitate novel macrofilaricidal drug discovery, we also undertook an integrated multidisciplinary study that leveraged our recent progress in the field of nematode genomics [40] and performed a systematic search for genes essential for the survival of filarial nematodes at a level not previously possible. Computational prioritization interfaced with experimental identification of repurposed drugs that are active against filarial nematodes resulted in a short list of prioritized targets and drug pairs. This was accomplished by first undertaking a target class repurposing approach and testing a library of compounds approved for clinical use (n = 2121, purchased by the Small Molecule Discovery Center, University of California San Francisco, CA) for their activity in vitro on adult *B. pahangi* female worms. Because one of the major challenges faced by investigators working in the field of discovery of drugs with activity against adult worms is the inability to screen large compound libraries and to follow up on the many new leads, we next intersected the actives with the omics-driven, computationally identified and prioritized target:drug pairs (n = 4442), which resulted in a short list of 25 prioritized drug candidates. We subsequently screened a subset of these prioritized drugs with adult worms the cattle filarial nematode *O. ochengi*. Finally, to demonstrate proof-of-concept, i.e., our ability to expand the chemical space and obtain supportive evidence for the identified potential target(s), we followed up on actives associated with two very different parasite protein target classes, thus establishing a solid platform for informed rational medicinal chemistry approaches in future studies. Those studies will lead to target validation and drug optimization, followed by in vivo activity confirmation using human equivalent dosages predicted based on jird pharmacokinetics data and human safety and clinical information. This successful approach can now also be used for identifying novel drugs and corresponding targets essential for the survival of other parasites, leveraging the extensive omics datasets for the human host.

## 2. Results and Discussion

### 2.1. Experimental Screening to Identify Active Macrofilaricidal Compounds Against Adult B. pahangi

**Figure 1:**
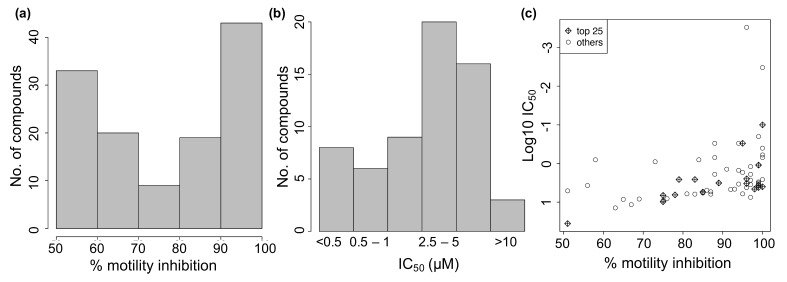
Effect of 124 repurposed drugs on *B. pahangi* motility (3-day assay). (**a**) Distribution of % motility inhibition for 124 active drugs. (**b**) IC_50_ values for 62 of the active drugs. (**c**) % motility inhibition vs IC_50_.

A library of 2121 drugs approved for clinical use (obtained from the UCSF Small Molecule Discovery Center) was screened in a phenotypic assay against adult female *B. pahangi* at 10 µM (Table S1) using the WormAssay software (University of California San Francisco, San Francisco, CA, USA) and dark-field plate imaging system [41]. A total of 124 drugs showed ≥50% inhibition of motility on day 3 (Figure 1A; Table S2) and a subset of 62 among these active drugs (based on motility inhibition and commercial availability) was screened in a dose response assay to determine their IC_50_ values (Figure 1B). The drugs showing higher levels of inhibition of motility tended to also have lower IC_50_ values (Figure 1C, correlation coefficient = −0.82 for the top hits).

### 2.2. Integrating Active Drugs with Computational Prioritization

**Figure 2:**
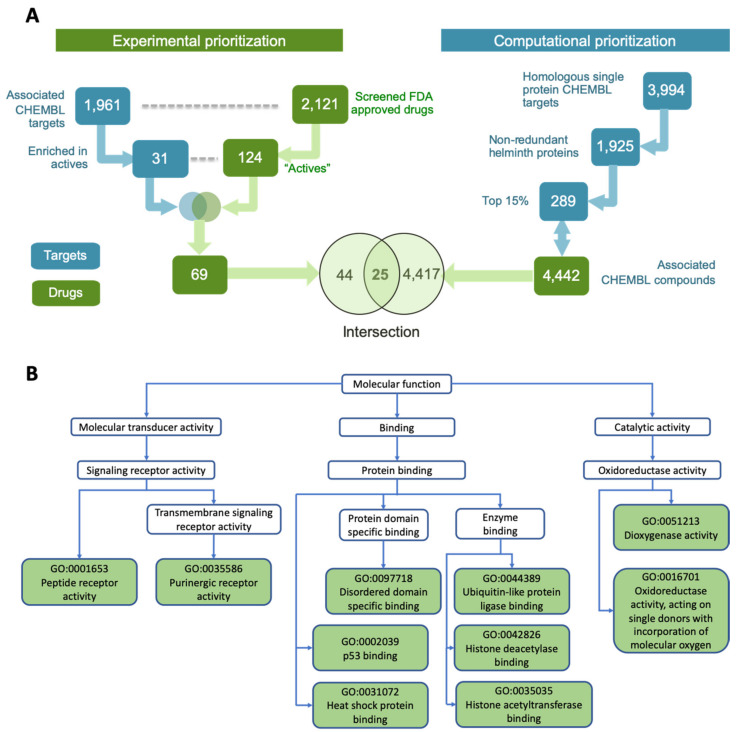
Identifying top hit compounds and functions associated with their targets. (**A**) Integrating experimental and computational prioritization schemes to obtain the top 25 candidate compounds. (**B**) Molecular functions represented by the 31 ChEMBL target proteins associated with the 124 active drugs.

The 2121 screened drugs were associated with 1961 potential targets in the ChEMBL database [42] (Figure 2A). Given the ‘many-to-many’ relationships between drugs and targets, we analyzed the 1961 potential ChEMBL targets to identify a subset with significant association with the 124 active drugs (as compared to the non-active screened drugs). This identified 31 targets showing significantly enriched association (Fisher’s exact test FDR-adjusted *p* ≤ 0.05) with the actives. These 31 targets showed functional association with multiple critical cellular processes (Figure 2B). These include receptor based signaling (e.g., adenosine, opioid, and chemokine receptors) [43,44,45], ubiquitin pathway (ubiquitin specific peptidase, ubiquitin conjugate enzyme) [46], transcription factors (e.g., p53, HIF1) [47,48] and oxidoreductase activity (e.g., ALOX12 and ALOX 15) [49]. These 31 targets were associated with 69 out of the 124 active drugs (Figure 2A).

Further prioritization among these 69 compounds was done by considering a set of potential anthelmintic targets previously reported by us, as a part of International Helminth Genomes Consortium [40]. Briefly, an omics-driven pipeline to identify the most promising targets from parasitic worms was implemented using 528,469 proteins from 33 parasitic worms (including all major filarial species of human importance), yielding identification of 3994 ChEMBL homologous targets (Figure 2A). Further reduction in redundancy, via recognition of members of the same orthologous protein families, reduced the 3994 targets to 1925 ChEMBL targets. Identifying the commercially available compounds associated with these top targets having desirable properties (e.g., Quantitative Estimate of Druglikeness (QED) score, FDA approved) and selecting a structurally diverse set among these resulted in 4442 drugs and drug-like compounds (Figure 2A). 

Intersecting the two parallel analyses from both computational and experimental prioritizations, resulted in a list of 25 overlapping hit compounds (Figure 2A). This set of drugs covered wide structural space (median pairwise Tanimoto similarity = 0.096) and is comprised of different compound classes, including benzyl ether azoles (antifungals targeting membrane sterol synthesis [50]), dihydropyridines (calcium channel blockers, for hypertension [51]), quinoline derivatives (antimalarials and antimicrobials [52]), and phenylpropanes (vasoactive drugs [53]). This high priority set of hits was then manually curated and further modified (for details *see* Methods), resulting in 18 prioritized hits that were experimentally effective in inhibiting motility (>50% inhibition) of adult *B. pahangi* at 10 μM (Table 1), with most having known putative targets [54,55,56,57,58,59,60,61,62,63,64,65,66,67,68,69,70,71,72,73,74,75,76,77,78,79]. 

**Figure 3:**
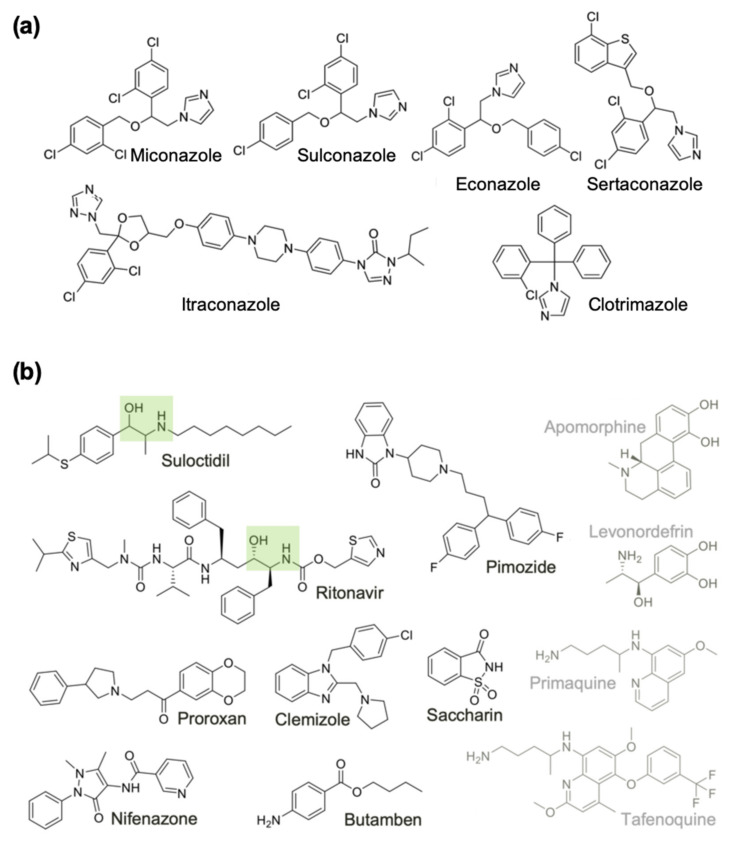
Chemical structures of the 18 active drugs on adult female *B. pahangi* divided into (**a**) Group A, and (**b**) Group B compounds. Compounds in Group B are classified into high priority because they are amenable to modifications (Black), or low priority due to central nervous system activity and due to unknown mode of action (Grey). The HEA moieties in two Group B compounds are highlighted with green boxes.

We noticed that many of the prioritized hits (6/18) were antifungal azoles. Henceforth we treated these azoles as belonging to a single group (Group A), separate from the remaining 12 compounds of diverse structures (Group B) (Figure 3). We further categorized the 12 Group B drugs based on the % motility inhibition into high (>80%), moderate (65–80%) and low motility inhibition (50–65%) group. Then, two drugs per each group were assayed to determine IC_50_. As expected, the lowest IC_50_ values were observed for the high % motility inhibition group, 2.5 and 3.7 μM (suloctidil and pimozide, respectively) and higher IC_50_ values for the other 2 groups (clemizole 6.5 μM, proroxan 8.5 μM, tafenoquine 14.1 μM and primaquine 5.1 μM). Compounds in Group B were classified into high priority because they are amenable to modifications, or low priority due to central nervous system (CNS) activity and due to unknown mode of action (Figure 3).

### 2.3. Screening of Prioritized Hits in Other Filarial Species

Our ultimate goal is to identify drugs with macrofilaricidal activity against the human parasite *O. volvulus*. One of the major challenges faced by investigators working in the field of drug discovery and neglected parasitic diseases is the ability to screen large compound libraries to identify new leads for the treatment of these highly prevalent, debilitating diseases. In this study we employed a multi-faceted screening funnel that utilizes several species of filarial nematodes and their relevant life history stages, in conjunction with a chemogenomic approach to identify new leads. Since the parasite that causes onchocerciasis only infects humans, several closely related surrogate species are used in the preclinical studies, including a primary screen with *B. pahangi* (laboratory species) and a secondary screen with the surrogate species *O. ochengi*. *O. ochengi* infects cow and has been used for various in vivo intervention studies [80]. As *O. ochengi* adult worms are readily accessible from West African infected cattle, we used this screen as a useful secondary screen that can establish proof-of-principle for potential therapeutics for human onchocerciasis. A tertiary assay with the target species *O. volvulus* (larval and pre-adult worms maintained in vitro) and a counter screen with *Loa loa* (from human volunteers) add to the robustness of the screening program.

Following our screening funnel, the Group A drugs and the top 3 Group B drugs (prioritized based on IC_50_ < 5.1 µM with adult female *B. pahangi*) were screened against adult female (viability) and male (motility) *O. ochengi* worms in vitro. None of the Group A compounds strongly affect (>70%) the viability of female *O. ochengi* (7-day assay), but 2 of the 3 Group B drugs (suloctidil and primaquine) were highly effective on female *O. ochengi* (>87% inhibition of viability); the IC_50_ values for these two Group B drugs were 4.1 and 1.3 µM, respectively (Table 2). In comparison, 3 out of the 6 Group A compounds (miconazole, sulconazole, and clotrimazole) were highly effective (> 99% motility inhibition) on male *O. ochengi* (5-day assay), and all 3 Group B compounds (suloctidil, pimozide and primaquine) also showed potent activity against male *O. ochengi* worms (>96% motility inhibition; Table 2), with IC_50_ values of 5.5 µM and 0.4 µM for suloctidil and primaquine, respectively. It is possible that this stage-specific difference is partly a result of differences in assay conditions between male and female worms, i.e., the treated female worms reside in the worm masses, while the males exit the worm masses and are exposed to the media, hence are possibly more directly exposed to the drugs.

The screening results showed that both suloctidil and primaquine were potent across the *B. pahangi* and *O. ochengi* species, including both female and male *O. ochengi*. Thus, the two compounds were also screened against an advanced developmental stage of pre-adult *O. volvulus* worms (L5 larval stage [39]), and counter-screened against *L. loa* microfilariae (Table 2). Both the compounds at 10 µM were effective on OvL5, showing 100% inhibition of motility already on day 21. The counter-screen with *L. loa* is crucial in order to avoid potential severe adverse effects that are known to result from drugs that target mf, such as ivermectin, when used by individuals co-infected with a high load of *Loa loa* mf [31,81]. It is therefore an important strategy to ensure that the potential macrofilaricidal drugs are inactive against *L. Loa* mf. While both the drugs retained high activity against *O. volvulus* L5, primaquine proved to be more inactive against *L. loa* mf (no detectable inhibition on day 1 and inhibition of motility slowly increased to 50% on day 5 at 10 µM, IC_50_ = 18.5 µM) as compared to suloctidil (100% inhibition already on day 1 at 10 µM, IC_50_ = 4.1 µM) (Table 2). A lack of commercial macrofilaricides makes it difficult to put these results in translational perspective for human use and further investigations to determine the pharmacokinetics and pharmacodynamics are also needed to assess the potency of these drugs in vivo. However, two drugs that are on the DNDi (The Drugs for Neglected Diseases initiative) portfolio as macrofilaricidal candidates, oxfendazole and emodepside, [82,83] have similar IC_50_ values against filarial species. The anthelmintic drug oxfendazole, which is currently used in veterinary medicine, was reported to have IC_50_ of 7.6 µM (day 14 in culture) and 28.6 µM (day 19 in culture) at inhibiting motility of *O. volvulus* L5 larvae [84] and emodepside, another veterinary anthelmintic, has been reported to have a submicromolar motility inhibition IC_50_ (0.8 µM) in adult female *B. malayi* [85].

In summary, our results provide a proof-of-concept that targeting filarial-conserved genes essential for adult worm survival is predictive of anti-filaricidal activity across species, and that differential selectivity against *Brugia* and *Onchocerca* compared to *Loa* can be detected, indicating that selective targeting can be achieved.

### 2.4. Expanding the List of Potential Anti-Macrofilarial Drugs by Focusing on Two Specific Classes

To further expand the list of hit drugs that could have macrofilaricidal activity, two new alternative approaches were undertaken: expanding the most successful compound class (azoles) and exploring a promising target class (aspartic proteases). 

Azoles (Group A hit drugs), the most potent compound class in the *B. pahangi* adult assays, was expanded by screening additional compounds from the same class. Azoles had potent macrofilaricidal effects and have shared activities, i.e., they inhibit sterol demethylases (specifically 14-alpha demethylase) and are known antifungal agents [86] that act by inhibiting ergosterol synthesis which is needed for fungal membrane permeability. However, it is likely that these compounds have a different mechanism of action in Nematoda since they appear to lack an ortholog of this 14-alpha demethylase. Azoles have also been reported to inhibit other crucial genes like P-gp, multiple CYP proteins, certain ion channels and receptors, thromboxane synthase (an inflammation mediator), FYN (a Src family tyrosine kinase oncogene), heme oxygenase (heme metabolism; maintaining homeostasis under oxidative and other stresses), Indoleamine 2,3-dioxygenase (heme containing immunomodulator), etc., [87,88]. They have also been identified in our previous work as potential broad-spectrum anthelmintics [89] based on their known activity against malate dehydrogenase (MDH) [90,91]. One concern is that some of these drugs may exhibit poor water solubility and oral bioavailability and may also have unacceptable side-effects on oral administration. However, this is no longer an issue with second and third generation azoles (itraconazole, posaconazole, etc.) [92]. We decided to focus on this group and expand this set of compounds by including all active azoles from our primary screening. A total of 9 azole hits were identified (out of 49 screened; Table S3), all with % motility inhibition of >80%: 6 from our integrated hits, 1 from the primary screen (tioconazole), and 3 from the ReFRAME library (a library of repurposed drugs) [93] (fenticonazole, isoconazole and posaconazole) (Table 2). For 7 of the 10 azoles, IC_50_ values for adult female *B. pahangi* ranged from 1.1 to 5.5 µM. When tested in adult female *O. ochengi*, 1 of these 10 azoles inhibited female worm viability by over 50% (isoconazole), compared to 7 azoles inhibiting adult male worm motility greater than 75%. For female *B. pahangi*, the IC_50_ values for fenticonazole, isoconazole, and posaconazole were 1.2, 3.8 and 0.1 µM, respectively; for female *O. ochengi*, these compounds lacked high potency, with IC_50_ values of 95, 31, and >100 µM, respectively, whereas the potency in male *O. ochengi* was better with IC_50_ values of 10.6, 0.6, and 6.1 µM respectively (Table 2). In *O. volvulus* L5, the IC_50_ for the fenticonazole and isoconazole were 4.7 and 2.4 µM, respectively. 

**Figure 4:**
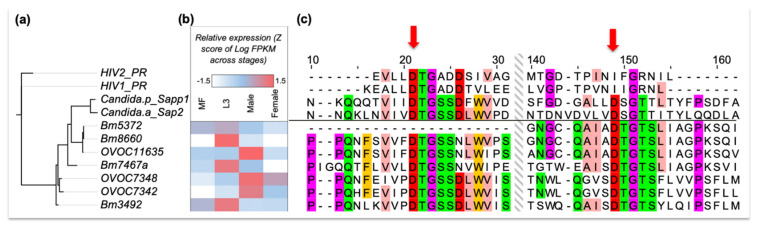
(**a**) A maximum-likelihood phylogenetic tree based on sequence similarity of aspartic proteases in *B. malayi, O. volvulus,* HIV and two *Candida* species. (**b**) Developmental stage-specific (mf, L3, and male or female adult worms) gene expression levels of the filarial APs in *B. malayi* and *O. volvulus*, as indicated by their protein IDs starting with Bm and OVOC, respectively. (**c**) Amino acid sequence alignments of aspartic proteases (shown only around the DTG/DTG and DTG/DSG motifs), with the two aspartic acid residues in the active site indicated by red arrows. HIV-PR only uses a single DTG active site as it forms a homodimer to constitute its protease activity. The alignment is color coded using the Zappo color scheme of Jalview, colors the residues according to their physicochemical properties—aliphatic or hydrophobic (pink), orange (aromatic), red (negative), green (hydrophilic), purple (conformationally special). Only the residues with moderate or high conservation are colored (i.e., conserved in at least 5 out of 11 aligned residues).

In addition to this class of drugs, we also focused on aspartic proteases (APs) as potential targets, using known aspartic protease inhibitors (APIs) [94,95]. This was based on our observation that one of our hit drugs, suloctidil, that showed consistently high potency across species and worm gender contains a hydroxyethylamine (HEA) functional group, and it has an overall substructure similar to ritonavir, a known API that is also among our top 18 candidate drugs (Figure 3B). Ritonavir is an inexpensive HEA group containing inhibitor of the aspartyl HIV-protease (HIV-PR) and is on the WHO list of essential medicines for its antiretroviral activity against HIV and AIDS [96]. Interestingly, levonordefrin, another hit drug (Table 1), also contains the HEA substructure. It is plausible that these three compounds might be targeting one or more of the *Brugia* aspartic proteases (Figure 4). APs are attractive targets due to their crucial functions in helminths [97], and they have been well studied in various parasitic nematode species [98,99,100], including filarial worms [101].

Protease inhibitors in general, and APIs in particular, have previously been developed as therapies for various diseases, e.g., hypertension, cancer, malaria, Alzheimer’s, and AIDS [102], with 5–10% of all drugs under development targeting proteases [103]. For APIs, this includes drugs designed using computational approaches, to specifically mimic the transition state, often via a HEA moiety or ‘warhead’ forming key H-bonding electrostatic contacts at the active site in the ‘oxyanion hole’, analogous to the substrate tetrahedral intermediate upon addition of water to the amide bond. APIs are especially amenable for in silico de novo design because APs form a tetrahedral substrate transition state with no covalent bonds (in contrast to serine or cysteine proteases) between the enzyme and the substrate and that can be mimicked by rationally designed molecules. Examples of such drugs include saquinavir and ritonavir, which were identified as potent inhibitors of HIV-PR. Interestingly, they have also been shown to be effective against other parasitic species, presumably targeting their homologous APs. For example, ritonavir is known to inhibit pathogenic *Candida* species [104], and was shown to dock in the *Trypanosoma cruzi* AP active site [105]. In some species, including HIV, the active site is reconstituted by homodimerization of the protein and requires two critical DTG or DSG active motifs, one from each monomer. We identified 11 APs in *B. malayi*, 8 in *B. pahangi* and 17 in *O. volvulus* using sequence-similarity against the MEROPS peptidase database [106] (BLASTP E-value <10^−4^). Among these, 4 in *B. malayi* and 3 in *O. volvulus* have conserved DTG motifs, suggesting that these are potential targets of ritonavir-like APIs (Figure 4). Unlike HIV, but similar to many other organisms (e.g., humans), the nematode aspartic proteases have an active site that appears to be part of the same protein and does not need dimerization. Multiple AP gene copies might result in functional redundancy (notably *B. pahangi*, the species used for primary screening, also has 3 orthologs—BPAG_0001244901, BPAG_0001278201 and BPAG_0000957001—for these 4 APs in *B. malayi*). However, the different stage-specific expression profiles (Figure 4) suggest that these may be crucial genes in different life cycle stages, including the developmental stages inhabiting the human host, e.g., mf, L3, L4, and adults. Specifically, Bm8660 and OVOC11635, which are the proteins most closely related to human Cathepsin D (CD). OVOC11635 shares 94% identity over the full length with previously characterized *O. volvulus* AP (cathepsin D [101]), and shows significant overexpression in L3 and adult male, respectively. CD is a more broadly active AP than HIV PR, and has multiple crucial physiological functions in vertebrates, e.g., metabolic degradation of intracellular proteins, activation of enzyme precursors, and regulation of apoptosis [107]. In Nematoda, CDs can function in invasion of host tissue, modulating host immune response and digestion of host proteins. In *C. elegans*, CD is the most significant enzyme for substrate macromolecular digestion in vitro [108].

In addition to the APIs included in our library of approved drugs for clinical use, 9 other drugs that inhibit HIV-PR are FDA-approved [109]. We were able to purchase 5 of these 9 APIs, and also purchased aliskiren (an FDA approved renin inhibitor) and pepstatin A (a highly potent general AP inhibitor not approved for clinical usage). The 7 APIs were screened against *B. pahangi* adult females, adult *O. ochengi* male and female worms, and *O. volvulus* L4 larvae using motility assays, to test their possible stage-specific efficacy (since some of the filarial aspartic proteases have stage-specific expression [110] (Figure 4B). Two of the HIV-PR inhibitors, nelfinavir and lopinavir (30 µM), inhibited the motility of female *B. pahangi* by >98% (Table 3), male *O. ochengi* by >88%, and *O. volvulus* L4 by >56%. In comparison, nelfinavir was highly effective against female *O. ochengi* viability (100%), while lopinavir was not effective at all against *O. ochengi* female worms, which might indicate a differential stage-specific activity. Pepstatin A showed species-specific activity, i.e., was only active against *O. ochengi*, with preferential activity against male (100% motility inhibition) compared to female (50% viability inhibition) worms. Higher transcriptional expression of Cathepsin D in *Onchocerca* male compared to female worms (Figure 4B) may partially explain this observation. In addition, cuticle permeability may be also determining the differential activity since suloctidil, which is one of our hits and was one of the factors in our decision to explore APs as targets of interest, along with 2 of our API hits (nelfinavir and lopinavir) have significantly higher cLogP values compared to the 6 inactive (6.0 vs. 3.8, P = 8.9 × 10^−4^, two-tailed T-test).

Ritonavir and the 7 APIs mentioned above were also assayed using a molting assay, observing the extent to which the of *O. volvulus* L3 to L4 larval molting, when incubated with the compounds is affected. All the APIs, except for ritonavir and atazanavir, showed moderate percentage molting inhibition on day 6 (≥40%) with 10 µM dosage. This means that some of the APIs, including darunavir, aliskiren and amprenavir, demonstrate a stage specific activity. This could potentially be related to developmental stage-based expression variation of some APs, the putative targets of these compounds (Figure 4B), with mid-to-high level expression (i.e., positive stagewise Z-scores) in the L3 larval stage with especially high expression seen in *B. malayi* L3. However, nelfinavir and lopinavir show activity in almost all the stages and species assayed (lopinavir was not effective at 30 µM on *O. ochengi* female worms) (Table 3). Interestingly, when we compared the IC_50_ values, the vasodilator suloctidil was more potent against adult *O. ochengi* (IC_50_ 4.1 µM for female and 5.5 µM for male) than ritonavir (IC_50_ 27.7 µM and 15.9 µM, in male and female worms, respectively), nelfinavir (IC_50_ 19 µM in both male and female worms) and lopinavir (IC_50_ 27 µM in male; not determined in female worms, >100 µM). Nonetheless, these IC_50_ values for these APIs are within the range of oxfendazole (veterinary anthelmintic in clinical trial for human onchocerciasis [82,83,84]) making them a great starting target class for optimization. It is notable, that even though these APIs consistently show only moderate activity in female *O. ochengi* worms, these worms are quite susceptible to other drugs, including our positive control for these experiments, Auranofin, a potent thioredoxin reductase inhibitor [111], with 100% loss of viability (10 µM) in all such assays. Nevertheless, these preliminary data spanning multiple filarial species and developmental stages suggest that APIs are effective in inhibiting the natural worm enzyme(s) and are excellent leads for expanding the chemical space as chemoprophylactic drugs as well as for macrofilaricidal drugs.

**Figure 5:**
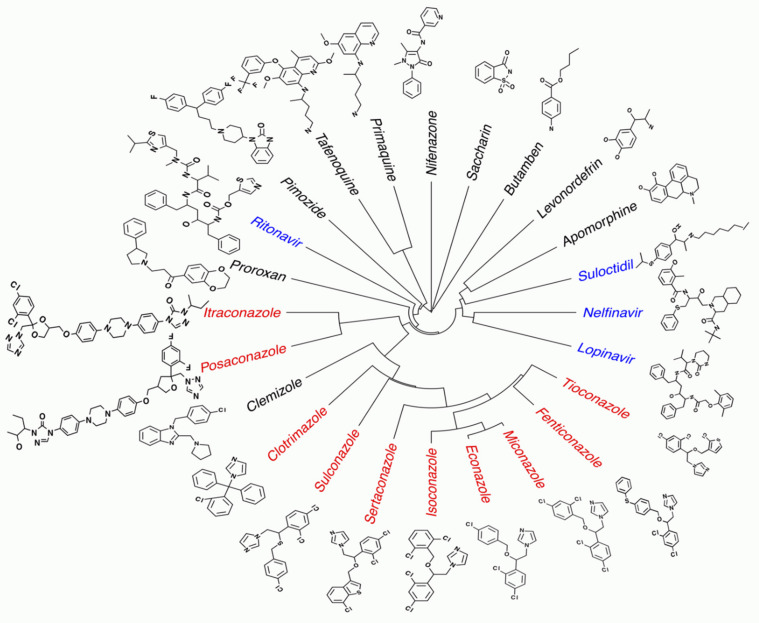
Structural similarity of the hit drugs identified in the primary and secondary phenotypic in vitro screen. Hits: at least 50% motility inhibition at 10 μM or 30 μM drug treatment of either female *B. pahangi*, or viability (female) and motility (male) inhibition of *O. ochengi*, or motility inhibition of *O. volvulus* L4. Red font: Azoles (Group A from primary screening hits and active azoles from expanded set). Blue font: active aspartic protease inhibitors and HEA containing compounds from primary screening hits.

In summary, our primary and secondary phenotypic in vitro screens have identified 24 drugs with potential macrofilaricidal activity, of which 13 were hits in at least 2 filarial species, and 7 showed gender-specific activity. Significantly, one of the tested hit drugs was also active against the *Brugia* and *Onchocerca* adult stages but more inactive against the *L. loa* mf, which is required for drug use in *L. loa* co-endemic regions. While we followed up on two compound classes, the azoles and the APIs, other hits are equally intriguing. For example, pimozide, proroxan, and clemizole, which have high anthelminthic potency against *B. pahangi* (IC_50_ of 2.5, 8.5, and 6.5 µM, respectively) are structurally similar CNS-active drugs that target different GPCRs. Pimozide and clemizole are benzimidazoles known to be dopamine [56,57] and histamine antagonists [60], respectively, but also have other related targets [58,61], and proroxan is a known adrenergic blocker. Overall, the hit drugs identified in this study present a broad range of structural space that can be explored further (Figure 5).

## 3. Conclusions

Overall, our integrated computational and experimental in vitro screening approach has proven to be highly successful for identifying new early leads and their putative parasite targets, while also confirming their essentiality for filarial adult worm fitness in phenotypic in vitro assays. Phenotypic in vitro drug screening based on motility of adult filarial worms has been extensively used to assess the potency of various chemical classes on filarial worm viability [84,85,112,113,114,115]. This study, however, not only undertook multiple screening assays in different species, sex and developmental stages of filarial worms adding rigor to the identification of potential drugs, but also using the computational analyses has subsequently allowed us to prioritize and expand the set of promising target and drug paired compounds (azoles and APIs) to show broad pan-filarial anti-macrofilarial activity. With good anti-macrofilarial potency, high potential for structural optimization, and known canonical therapeutic targets these hits offer a promising starting point for identifying lead drug scaffolds and targets for optimizing and characterizing novel anti-filarial drugs. Once future studies validate these filarial parasite targets, these can be further used for selective drug optimization and followed by in vivo activity confirmation using human equivalent dosages predicted based on animal PK data and human safety and clinical information. We posit that this successful approach can now be also used for identifying novel drugs and corresponding targets essential for the survival of other parasites, leveraging the extensive omics datasets for the human host.

## 4. Materials and Methods

### 4.1. Ethics Statement

*L. loa* mf were collected from adult patient donors, aged 21 or older, with 2000+ mf per mL of blood living in the Edea Health District of the Littoral Region of Cameroon. Each patient provided written and signed informed consent, and ethical and administrative approval was obtained from the Cameroon National Ethics Committee (N°2013/11/371/L/CNERSH/SP) and the Cameroon Ministry of Health.

Adult female *B. pahangi* were collected from jirds (*Meriones unguiculatus*) at TRS labs Inc., Athens, Georgia and from the Univ. of Missouri–Columbia Institutional Animal Care Facility (IACUC approval #8623 and #9537).

### 4.2. Experimental Screening of a Library of Drugs Approved for Clinical Use Against B. pahangi

Individual adult female *B. pahangi* were placed in media (RPMI-1640 with 25 mM HEPES, 2.0 g/L NaHCO_3_, 5% heat inactivated fetal bovine serum (FBS), and 1X Antibiotic and Antimycotic solution) in 24-well plates. A Biomek FxP liquid handler was used to remove excess media so that each well contained one female worm in 500 µL media. Compounds dissolved in DMSO were added to each well at a concentration of 10 µM, with 4 replicates per compound and 1% DMSO was used as a vehicle control and 10 µM Auranofin as a positive control. Cultures were maintained in a 37 °C incubator over the course of the assay, and motility measurements were recorded using the WormAssay software [116], dark-field plate recording apparatus and 1080p digital camcorder on days 0–3. Compounds that caused 75% or greater inhibition of motility by day 3 were considered hits and were then tested in IC_50_ assays, using a 6-point serial dilution ranging from 30 µM to 0.1 µM. IC_50_ values were calculated using GraphPad Prism (GraphPad Software, San Diego, CA), and this data was used to prioritize compounds for further in vitro screening with *Onchocerca spp.* and *L. loa* microfilaria. Selected compounds (i.e., the aspartyl protease inhibitors) were screened at 30 µM and an additional motility reading was recorded on day 6.

### 4.3. Experimental Screening of Prioritized Drugs in O. ochengi Adults

Nodules containing adult *O. ochengi* were collected from the umbilical skin of infected cows from abattoirs in Douala, Cameroon. Worm masses containing one adult female and 0–7 adult males were excised from the nodules and incubated in 4 mL of complete *Onchocerca* culture medium (RPMI-1640, 5% newborn calf serum, 200 units/mL penicillin, 200 μg/mL streptomycin and 2.5 μg/mL amphotericin B (Sigma–Aldrich)) in 12-well culture plates. Worm masses were incubated at 37 °C, 5% CO_2_ overnight to allow adult males to egress from the mass into the surrounding media. Compounds were then added to wells at a final concentration of 10 µM for initial screening; 1% DMSO was used as a negative control. Testing was conducted as previously described [117] in quadruplicate in each assay, and assays were conducted twice on separate days to ensure reproducibility. On day 5, after compound addition, inhibition of male motility was scored according to the following criteria: 100% (complete inhibition of motility), 90% (only head or tail of worm moving or vibrating), 75% (worm very sluggish), 50% (worm sluggish), 25% (little change in motility), to 0% (no observable reduction in motility). On day 7, female viability was assessed using an MTT/formazan assay in which each worm mass was washed in PBS and then transferred to a 48-well plate with 500 μL of 0.5 mg/mL MTT (Sigma–Aldrich) in incomplete culture medium, and then incubated in the dark at 37^o^ C protected from light for 30 minutes. After incubation, female worm masses were visually examined and scored to determine inhibition of formazan production, with higher percent inhibition of formazan indicating reduced worm viability as previously described [117]. To calculate the IC_50_, quadruplicate worm masses were incubated using a 7-point serial dilution ranging from 30 µM to 0.03 µM and assays were conducted as described above. GraphPad Prism was used to calculate IC_50_ values.

### 4.4. Counter Screening Compounds with L. loa Microfilariae in Vitro

Ten mL of venous blood in an EDTA tube provided by each consented donor was layered on a stepwise 46 and 43% Percoll (GE Healthcare, Piscataway, NJ, USA) gradient, and then centrifuged at 400 rcf for 20 minutes. *L. loa* mf were collected from the 43% layer and washed 3× in complete culture media. *Loa* mf were then cultured in 96-well plates (10–15 per well) containing a confluent layer of monkey kidney epithelial cells as previously described by Cho-Ngwa et al. (2010) [117]. Compounds were tested at 10 µM in duplicate wells and 10 μg/mL ivermectin was used as a positive control. Motility was scored daily for 5 days after compound addition using the same scale as the motility of *O. ochengi* adult males.

### 4.5. O. volvulus L5 Motility and Viability Assay

High priority compounds, i.e., those that had previously shown activity against *Onchocerca spp.* and *Brugia spp.*, were tested with *O. volvulus* L5 in vitro. L3 larvae were cultured to L5 larvae as described by Voronin et al., 2019 [39]. Once reaching the pre-adult and L5 stage after at least 70 days in culture, L5 were placed in transwell inserts in a 24-well plate and treated with drugs, as previously described [39]. Briefly, each transwell insert was added to a well containing 500 µL OvL4-CMS media with a human umbilical vein endothelial cell (HUVEC) monolayer, and 8–10 L5 were cultured in each transwell insert. Compounds were tested in triplicate, with the concentration selected based on the potency found in *B. pahangi* in vitro assays. OvL5 were treated for 14 days and then in normal media as described [39]. Inhibition of motility was scored on a 0–100% scale continuously (every 2–3 days) throughout the 28-day assay. Beyond 28 days the control worms also start showing loss of motility, thus the 28 day was selected as an end point. For some (suloctidil and primaquine) drugs, the effectiveness was already highly evident by day 21, obviating the need to continue the assay beyond that point. Viability was measured at the end of the motility assay (day 29) by incubating the L5s with 0.1% MTT in PBS at 37 °C for 1 h, then visually examining the larvae for formazan production as indicated by blue coloration of the worm. If less than 50% of the worm was stained blue the worm was considered dead, and if greater than 50% of the worm was stained blue the worm was considered alive.

### 4.6. O. volvulus L4 Motility Assay

L4 (day 9 after molting) were cultured with 30 µM of the 7 APIs, and the motility of the L4 was monitored over 6 days. The anthelmintic flubendazole at 10 µM was used as a positive control.

### 4.7. Computational Identification and Prioritization of Targets and Inhibitors

Our starting step was to identify genes essential for filarial nematode survival by bioinformatically parsing the recently published database of genes essential for helminth survival [40]. That database of 81 helminth genomes was published by the International Helminth Genomes Consortium, and included a systematic search for genes essential for helminth survival [40]. In short, a target score was assigned to each of these worm proteins to quantify their likely quality as targets based on prior knowledge about their advantageous or disadvantageous properties: (i) the quality (E-value and target coverage) of the BLASTP match between the helminth and ChEMBL target proteins; (ii) whether the ChEMBL target had a close human BLAST match (since targeting a protein that lacks a human homolog is less likely to cause undesirable side-effects); (iii) whether the helminth gene had a *C. elegans* or *D. melanogaster* homolog with a severe phenotype (for example, a lethal or sterile phenotype; (iv) whether it was expressed in key life cycle stages (for example, adult); (v) whether it had homologs in most members of a major helminth clade (for example, in most nematodes); whether it lacked within-species paralogs; whether it belonged to a Compara family with a highly conserved alignment; (vi) whether the matching ChEMBL protein had a structure in the PDBe; and (vii) whether it was from a non-chordate animal.

The compounds linked to the helminth targets were ranked based on: (i) QED score [118] (a score that quantifies a compound’s drug-likeness by integrating some relevant molecular properties together and correlates with its likelihood to become a hit); (ii) at least phase III approval of the compound as a drug (since it is expected to be quicker and more efficient and economical to develop a drug which has already passed early phase clinical trials); (iii) whether the compound (if an approved drug) could be administered orally or topically; and (iv) lack of serious side effects or predicted toxicology targets. A total of 4442 compounds were associated with the top 15% (289) of the potential targets.

To identify potential targets of the 124 active compounds their association with putative targets in the ChEMBL database was analyzed as follows: The 2121 screened compounds included 1705 compounds that could be identified in the ChEMBL database using various forms or synonyms of the common names for these compounds. These 1705 ChEMBL compounds were associated with 1961 potential ChEMBL targets (i.e., had a ChEMBL assay recorded with pchembl value >5, corresponding to 10 µM target-compound half-maximal response metric—e.g., IC_50_, EC_50_, and K_d_). Given the many-to-many relationships between compounds and targets, we analyzed the 1961 potential ChEMBL targets to identify a subset with significant association with the 124 active compounds. Out of the 1961 targets, 31 showed significantly enriched association (Fisher’s exact test FDR-adjusted *p* ≤ 0.05) with the active ChEMBL compounds. These 31 targets were associated (i.e., had ChEMBL records with pchembl >5) with 69 out of the 124 active compounds.

An intersection of these 69 drugs with anti-macrofilaricidal activity and the 4442 computational prioritized compounds resulted in 25 prioritized candidates for further evaluation. A subset of these compounds (13/25) was removed due to factors such as known issues with cytotoxicity and not being amenable to chemical modifications. Additionally, 6 other active drugs (from the 124 from the primary screen) were included in the final prioritized hits based on prior knowledge and literature support yielding the final list of 18 active drugs.

### 4.8. Expanding the List of Azoles as Specific Drug Class with Anti-Macrofilarial Potency

The azole class of compounds were initially identified from the library of drugs identified for clinical use obtained by the Small Molecule Discovery Center, University of California San Francisco, CA, as hits in the adult *Brugia* worm assay. Additional azoles were obtained from the Repurposing, Focused Rescue, and Accelerated Medchem (ReFRAME) library, which was generated by Calibr at Scripps Research, La Jolla, CA with support from the Bill and Melinda Gates Foundation. The ReFRAME library is a set of approximately 13,000 compounds that include FDA-approved or registered drugs, investigational drugs that are currently or have been tested previously in clinical phase trials [93].

### 4.9. Expanding the List of Aspartic Protease Inhibitors

The five FDA approved drugs that inhibit HIV-PR were identified from literature [109], aliskiren was included as an FDA approved renin inhibitor and pepstatin A was included due to being a highly potent general API not approved for clinical usage. APs in filarial worms were identified using orthology with known *C. elegans* APs, as annotated on Wormbase [119]. The orthologs were identified using Orthofinder [120]. Multiple sequence alignment was done using MAFFT [121], and visualized using Jalview [122]. The phylogenetic tree was estimated using PhyML 3.0 [123] and the node support values are calculated using an “aLRT SH-like” option. The developmental stage expression level values are obtained from [110].

### 4.10. Clustering of Hits Based on Structural Similarity

The clustering was based on (1—Tanimoto similarity measure) as distance metric, calculated using ChemmineR [124] package, and agglomerated using “complete linkage” method.

## Figures and Tables

**Table 1:** The 18 top hits (female *Brugia pahangi*, 3-day assay, >50% inhibition of motility and IC_50_ values from a subset of the hits).

Drug Common Name		Compound Class	Compound Subclass	Literature Targets (Not All are Listed)	*Brugia pahangi* (Adult Female)	
					% Motility Inhibition (10 μM)	IC_50_ (μM)
**Group A**	Econazole	Imidazole	Benzyl ether	Sterol 14-demethylase/K+ VGIC [72]	100	4.0
	Miconazole	Imidazole	Benzyl ether	Sterol 14-demethylase/K+ VGIC [72]	96	3.3
	Sulconazole	Imidazole	Thiobenzyl ether	Sterol 14-demethylase [73]	98	4.6
	Sertaconazole	Imidazole	Benzothiophene	Sterol 14-demethylase [74]	100	-
	Clotrimazole	Imidazole	Triphenylmethane	Sterol 14-demethylase/K+ VGIC [75]	85	5.5
	Itraconazole	Triazole	Piperazinyl	XIAP/K+ VGIC [76,77]	80	-
**Group B**	Suloctidil	Amino alcohol	Phenyl thioether	K+ VGIC/thyroid receptor [52,53]	99	3.7
	Pimozide	Benzimidazolone	Piperidine	DRD2/DRD3 (GPCRs)/KCNH2/Calmodulin [54,55,56]	96	2.5
	Butamben	Benzoic ester	Aniline	VGICs [57]	66	-
	Clemizole	Benzimidazole	Pyrrolidine	HRH1/K+ VGIC/NR ROR-gamma [58,59]	78	6.5
	Proroxan	Phenyl ketone	Phenyl pyrrolidine	Adrenergic receptors [60]	65	8.5
	Saccharin	Benzoisothiazolone	Sulfonyl amide	carbonic anhydrase [61]	57	-
	Ritonavir	Amino alcohol	Peptidomimetic	HIV protease/CYP3A/SLC47 [62,63,64]	52	-
	Nifenazone	Dihydropyrazole	Nicotinamide	-	50	-
	Levonordefrin	Amino alcohol	Phenol	ADRA2/HADH2/TF HIF1A/APE1 [65,66]	54	-
	Tafenoquine	Quinoline	Alkyl amine	cytochrome c reductase [67]	63	14.1
	Primaquine	Quinoline	Alkyl amine	quinone reductase [68,69]	51	5.1
	Apomorphine	Aporphine	Tetrahydroisoquinoline	Dopamine/serotonin/adrenergic receptor agonist [70,71]	53	-

**Table 2:** Expanded list of Azoles and 3 best Group B hit drugs against multiple filarial species.

Drug Common Name		Day 3 Adult Female *B. pahangi*		Day 7 Adult Female *O. ochengi*		Day 5 Adult Male *O. ochengi*		Day 28 *O. volvulus* L5		Day 5 *Loa loa* mf	
		% Inhibition of Motility (10 μM)	IC_50_ (μM)	% Inhibition of Viability (10 μM)	IC_50_ (μM)	% Inhibition of Motility (10 μM)	IC_50_ (μM)	% Inhibition of Motility (10 μM)	IC_50_ (μM)	% Inhibition of Motility (10 μM)	IC_50_ (μM)
**Group A**	Isoconazole	100	3.8	57	30.8	100	0.6	96	2.4	33.3	10
	Fenticonazole	100	1.2	32	94.7	75	10.6	83	4.7	50	10
	Sertaconazole	100	-	21	-	31	-	-	-	-	-
	Econazole	100	4.0	22	-	26	-	-	-	-	-
	Tioconazole	99	3.2	14	-	100	-	-	-	-	-
	Sulconazole	98	4.6	26	-	99	-	-	-	-	-
	Miconazole	96	3.3	0	-	100	-	-	-	-	-
	Clotrimazole	85	5.5	8	-	100	-	-	-	-	-
	Posaconazole	82	0.1	38	>100	79	6.1	57 *	-	-	-
	Itraconazole	80	-	13	-	21	-	-	-	-	-
**Group B**	Suloctidil	99	3.7	87	4.1	100	5.5	100 **	-	100	4.1
	Pimozide	96	2.5	13	-	96	-	-	-	-	-
	Primaquine	51	5.1	100	1.3	100	0.4	100 **	-	50	18.5

* Screen was done at 1 µM; ** 100% effective on day 21 and 100% inhibition of viability as measured by MTT assay; -, not tested.

**Table 3:** Known aspartic protease inhibitors with anti-filarial activity.

Aspartyl Protease Inhibitors (APIs)	% Inhibition of Motility (30 µM)				% Inhibition of Molting (L3 to L4)	
	*B. pahangi*	*O. ochengi*		*O. volvulus*	*O. volvulus* (day 6)	
	Female (day 6)	Female (day 7) **	Male (day 5)	L4 (day 7)	3 μM	10 μM
Nelfinavir	99	100	100	73	50.2	45.5
Lopinavir	98	0	88	56	0	74.1
Ritonavir	52 *	63	100	0	0	0
Pepstatin A	0	50	100	0	0	40
Darunavir	0	30	ND	4.4	43.9	46.7
Aliskiren	0	6	ND	0	30.4	51.7
Amprenavir	24	0	17	8	0	43.5
Atazanavir	11	42	ND	30	0	0

* Screen was done at 10 µM; ** % viability inhibition as measured by MTT assay; ND, could not be determined.

## Data Availability

The data presented in this study are available in the manuscript main tables and Supplementary Tables.

## Supplementary Materials

The following are available online at https://www.mdpi.com/2076-0817/10/1/71/s1, Table S1: Motility inhibition percentage values and IC_50_ values for the primary screening against *Brugia pahangi*. Table S2: IC_50_ and motility inhibition percentage values for all species assayed., Table S3: Motility, viability and molting assays across multiple nematode parasite species for the expanded set of Azole compounds.

## References

[1] Taylor M.J., Hoerauf A., Bockarie M. (2010). Lymphatic filariasis and onchocerciasis. Lancet.

[2] Hoerauf A., Pfarr K., Mand S., Debrah A.Y., Specht S. (2011). Filariasis in Africa-treatment challenges and prospects. Clin. Microbiol. Infect..

[3] Lustigman S., Prichard R.K., Gazzinelli A., Grant W.N., Boatin B.A., McCarthy J.S., Basanez M.G. (2012). A research agenda for helminth diseases of humans: The problem of helminthiases. PLoS Negl. Trop. Dis..

[4] Prichard R.K., Basanez M.G., Boatin B.A., McCarthy J.S., Garcia H.H., Yang G.J., Sripa B., Lustigman S. (2012). A research agenda for helminth diseases of humans: Intervention for control and elimination. PLoS Negl. Trop. Dis..

[5] King C.L., Suamani J., Sanuku N., Cheng Y.C., Satofan S., Mancuso B., Goss C.W., Robinson L.J., Siba P.M., Weil G.J. (2018). A Trial of a Triple-Drug Treatment for Lymphatic Filariasis. N. Engl. J. Med..

[6] Weil G.J., Bogus J., Christian M., Dubray C., Djuardi Y., Fischer P.U., Goss C.W., Hardy M., Jambulingam P., King C.L. (2019). The safety of double- and triple-drug community mass drug administration for lymphatic filariasis: A multicenter, open-label, cluster-randomized study. PLoS Med..

[7] Irvine M.A., Stolk W.A., Smith M.E., Subramanian S., Singh B.K., Weil G.J., Michael E., Hollingsworth T.D. (2017). Effectiveness of a triple-drug regimen for global elimination of lymphatic filariasis: A modelling study. Lancet Infect. Dis..

[8] Milton P., Hamley J.I.D., Walker M., Basanez M.G. (2020). Moxidectin: An oral treatment for human onchocerciasis. Expert Rev. Anti Infect. Ther..

[9] Molyneux D.H., Bradley M., Hoerauf A., Kyelem D., Taylor M.J. (2003). Mass drug treatment for lymphatic filariasis and onchocerciasis. Trends Parasitol..

[10] Molyneux D.H., Taylor M.J. (2001). Current status and future prospects of the Global Lymphatic Filariasis Programme. Curr. Opin. Infect. Dis..

[11] Molyneux D.H., Hopkins A., Bradley M.H., Kelly-Hope L.A. (2014). Multidimensional complexities of filariasis control in an era of large-scale mass drug administration programmes: A can of worms. Parasites Vectors.

[12] Plaisier A.P., van Oortmarssen G.J., Remme J., Habbema J.D. (1991). The reproductive lifespan of *Onchocerca volvulus* in West African savanna. Acta Trop..

[13] WHO (2010). Global programme to eliminate lymphatic filariasis. Wkly Epidemiol. Rec..

[14] Ottesen E.A., Hooper P.J., Bradley M., Biswas G. (2008). The global programme to eliminate lymphatic filariasis: Health impact after 8 years. PLoS Negl. Trop. Dis..

[15] Chu B.K., Hooper P.J., Bradley M.H., McFarland D.A., Ottesen E.A. (2010). The economic benefits resulting from the first 8 years of the Global Programme to Eliminate Lymphatic Filariasis (2000–2007). PLoS Negl. Trop. Dis..

[16] Diawara L., Traore M.O., Badji A., Bissan Y., Doumbia K., Goita S.F., Konate L., Mounkoro K., Sarr M.D., Seck A.F. (2009). Feasibility of onchocerciasis elimination with ivermectin treatment in endemic foci in Africa: First evidence from studies in Mali and Senegal. PLoS Negl. Trop. Dis..

[17] Traore M.O., Sarr M.D., Badji A., Bissan Y., Diawara L., Doumbia K., Goita S.F., Konate L., Mounkoro K., Seck A.F. (2012). Proof-of-principle of onchocerciasis elimination with ivermectin treatment in endemic foci in Africa: Final results of a study in Mali and Senegal. PLoS Negl. Trop. Dis..

[18] NTD Modelling Consortium Onchocerciasis Group (2019). The World Health Organization 2030 goals for onchocerciasis: Insights and perspectives from mathematical modelling: NTD Modelling Consortium Onchocerciasis Group. Gates Open Res..

[19] NTD Modelling Consortium Lymphatic Filariasis Group (2019). The roadmap towards elimination of lymphatic filariasis by 2030: Insights from quantitative and mathematical modelling. Gates Open Res..

[20] Turner H.C., Churcher T.S., Walker M., Osei-Atweneboana M.Y., Prichard R.K., Basanez M.G. (2013). Uncertainty surrounding projections of the long-term impact of ivermectin treatment on human onchocerciasis. PLoS Negl. Trop. Dis..

[21] Turner H.C., Walker M., Churcher T.S., Osei-Atweneboana M.Y., Biritwum N.K., Hopkins A., Prichard R.K., Basanez M.G. (2014). Reaching the London Declaration on Neglected Tropical Diseases Goals for Onchocerciasis: An Economic Evaluation of Increasing the Frequency of Ivermectin Treatment in Africa. Clin. Infect. Dis. Off. Publ. Infect. Dis. Soc. Am..

[22] GBD 2015 Disease and Injury Incidence and Prevalence Collaborators (2016). Global, regional, and national incidence, prevalence, and years lived with disability for 310 diseases and injuries, 1990-2015: A systematic analysis for the Global Burden of Disease Study 2015. Lancet.

[23] Hopkins A.D. (2016). Neglected tropical diseases in Africa: A new paradigm. Int. Health.

[24] Kim Y.E., Remme J.H., Steinmann P., Stolk W.A., Roungou J.B., Tediosi F. (2015). Control, elimination, and eradication of river blindness: Scenarios, timelines, and ivermectin treatment needs in Africa. PLoS Negl. Trop. Dis..

[25] African Programme for Onchocerciasis Control (APOC) (2014). Report of the Thirty-Eight Session of the Technical Consultative Committee: Ouagadougou.

[26] The U.S. Food and Drug Administration (FDA), The United States Department of Agriculture (USDA) (2018). Drug Approval Package: Moxidectin.

[27] Awadzi K., Opoku N.O., Attah S.K., Lazdins-Helds J., Kuesel A.C. (2014). A randomized, single-ascending-dose, ivermectin-controlled, double-blind study of moxidectin in Onchocerca volvulus infection. PLoS Negl. Trop. Dis..

[28] Opoku N.O., Bakajika D.K., Kanza E.M., Howard H., Mambandu G.L., Nyathirombo A., Nigo M.M., Kasonia K., Masembe S.L., Mumbere M. (2018). Single dose moxidectin versus ivermectin for Onchocerca volvulus infection in Ghana, Liberia, and the Democratic Republic of the Congo: A randomised, controlled, double-blind phase 3 trial. Lancet.

[29] Turner H.C., Walker M., Attah S.K., Opoku N.O., Awadzi K., Kuesel A.C., Basanez M.G. (2015). The potential impact of moxidectin on onchocerciasis elimination in Africa: An economic evaluation based on the Phase II clinical trial data. Parasites Vectors.

[30] Kelly-Hope L.A., Cano J., Stanton M.C., Bockarie M.J., Molyneux D.H. (2014). Innovative tools for assessing risks for severe adverse events in areas of overlapping Loa loa and other filarial distributions: The application of micro-stratification mapping. Parasites Vectors.

[31] Boussinesq M., Gardon J., Gardon-Wendel N., Chippaux J.P. (2003). Clinical picture, epidemiology and outcome of *Loa*-associated serious adverse events related to mass ivermectin treatment of onchocerciasis in Cameroon. Filaria J..

[32] D’Ambrosio M.V., Bakalar M., Bennuru S., Reber C., Skandarajah A., Nilsson L., Switz N., Kamgno J., Pion S., Boussinesq M. (2015). Point-of-care quantification of blood-borne filarial parasites with a mobile phone microscope. Sci. Transl. Med..

[33] Boatin B.A., Basanez M.G., Prichard R.K., Awadzi K., Barakat R.M., Garcia H.H., Gazzinelli A., Grant W.N., McCarthy J.S., N’Goran E.K. (2012). A research agenda for helminth diseases of humans: Towards control and elimination. PLoS Negl. Trop. Dis..

[34] Osei-Atweneboana M.Y., Awadzi K., Attah S.K., Boakye D.A., Gyapong J.O., Prichard R.K. (2011). Phenotypic evidence of emerging ivermectin resistance in Onchocerca volvulus. PLoS Negl. Trop. Dis..

[35] Doyle S.R., Bourguinat C., Nana-Djeunga H.C., Kengne-Ouafo J.A., Pion S.D.S., Bopda J., Kamgno J., Wanji S., Che H., Kuesel A.C. (2017). Genome-wide analysis of ivermectin response by Onchocerca volvulus reveals that genetic drift and soft selective sweeps contribute to loss of drug sensitivity. PLoS Negl. Trop. Dis..

[36] Lustigman S., McCarter J.P. (2007). Ivermectin Resistance in *Onchocerca volvulus*: Toward a Genetic Basis. PLoS Negl. Trop. Dis..

[37] Debrah A.Y., Specht S., Klarmann-Schulz U., Batsa L., Mand S., Marfo-Debrekyei Y., Fimmers R., Dubben B., Kwarteng A., Osei-Atweneboana M. (2015). Doxycycline Leads to Sterility and Enhanced Killing of Female Onchocerca volvulus Worms in an Area with Persistent Microfilaridermia After Repeated Ivermectin Treatment: A Randomized, Placebo-Controlled, Double-Blind Trial. Clin. Infect. Dis. Off. Publ. Infect. Dis. Soc. Am..

[38] Veale C.G.L. (2019). Unpacking the Pathogen Box-An Open Source Tool for Fighting Neglected Tropical Disease. ChemMedChem.

[39] Voronin D., Tricoche N., Jawahar S., Shlossman M., Bulman C.A., Fischer C., Suderman M.T., Sakanari J.A., Lustigman S. (2019). Development of a preliminary in vitro drug screening assay based on a newly established culturing system for pre-adult fifth-stage Onchocerca volvulus worms. PLoS Negl. Trop. Dis..

[40] International Helminth Genomes Consortium (2019). Comparative genomics of the major parasitic worms. Nat. Genet..

[41] Marcellino C., Gut J., Lim K.C., Singh R., McKerrow J., Sakanari J. (2012). WormAssay: A novel computer application for whole-plate motion-based screening of macroscopic parasites. PLoS Negl. Trop. Dis..

[42] Mendez D., Gaulton A., Bento A.P., Chambers J., De Veij M., Felix E., Magarinos M.P., Mosquera J.F., Mutowo P., Nowotka M. (2019). ChEMBL: Towards direct deposition of bioassay data. Nucleic Acids Res..

[43] Sheth S., Brito R., Mukherjea D., Rybak L.P., Ramkumar V. (2014). Adenosine receptors: Expression, function and regulation. Int. J. Mol. Sci..

[44] Waldhoer M., Bartlett S.E., Whistler J.L. (2004). Opioid receptors. Annu. Rev. Biochem..

[45] Hughes C.E., Nibbs R.J.B. (2018). A guide to chemokines and their receptors. FEBS J..

[46] Kerscher O., Felberbaum R., Hochstrasser M. (2006). Modification of proteins by ubiquitin and ubiquitin-like proteins. Annu. Rev. Cell Dev. Biol..

[47] Kastenhuber E.R., Lowe S.W. (2017). Putting p53 in Context. Cell.

[48] Balamurugan K. (2016). HIF-1 at the crossroads of hypoxia, inflammation, and cancer. Int. J. Cancer.

[49] Mashima R., Okuyama T. (2015). The role of lipoxygenases in pathophysiology; new insights and future perspectives. Redox Biol..

[50] Sheehan D.J., Hitchcock C.A., Sibley C.M. (1999). Current and emerging azole antifungal agents. Clin. Microbiol. Rev..

[51] Epstein B.J., Vogel K., Palmer B.F. (2007). Dihydropyridine calcium channel antagonists in the management of hypertension. Drugs.

[52] Marella A., Tanwar O.P., Saha R., Ali M.R., Srivastava S., Akhter M., Shaquiquzzaman M., Alam M.M. (2013). Quinoline: A versatile heterocyclic. Saudi Pharm. J..

[53] Deliorman D., Calis I., Ergun F., Dogan B.S., Buharalioglu C.K., Kanzik I. (2000). Studies on the vascular effects of the fractions and phenolic compounds isolated from Viscum album ssp. album. J. Ethnopharmacol..

[54] Malaisse W.J. (1977). Calcium-antagonists and islet function X. Effect of suloctidie. Arch. Int. Pharmacodyn. Ther..

[55] Chatelain P., Reckinger N., Roncucci R. (1979). Effect of suloctidil on Na+/K+ ATPase activity and on membrane fluidity in rat brain synaptosomes. Biochem. Pharmacol..

[56] Chen X., Ji Z.L., Chen Y.Z. (2002). TTD: Therapeutic Target Database. Nucleic Acids Res..

[57] Wise L.D., Pattison I.C., Butler D.E., DeWald H.A., Lewis E.P., Lobbestael S.J., Nordin I.C., Poschel B.P., Coughenour L.L. (1985). 1-[3-(Diarylamino)propyl]piperidines and related compounds, potential antipsychotic agents with low cataleptogenic profiles. J. Med. Chem..

[58] Katagi J., Nakamura Y., Cao X., Ohara H., Honda A., Izumi-Nakaseko H., Ando K., Sugiyama A. (2016). Why Can dl-Sotalol Prolong the QT Interval In Vivo Despite Its Weak Inhibitory Effect on hERG K(+) Channels In Vitro? Electrophysiological and Pharmacokinetic Analysis with the Halothane-Anesthetized Guinea Pig Model. Cardiovasc. Toxicol..

[59] Bang S., Yang T.J., Yoo S., Heo T.H., Hwang S.W. (2012). Inhibition of sensory neuronal TRPs contributes to anti-nociception by butamben. Neurosci. Lett..

[60] Finkelstein M., Kromer C.M., Sweeney S.A., Delahunt C.S. (1960). Some aspects of the pharmacology of clemizole hydrochloride. J. Am. Pharm. Assoc..

[61] Richter J.M., Schaefer M., Hill K. (2014). Clemizole hydrochloride is a novel and potent inhibitor of transient receptor potential channel TRPC5. Mol. Pharmacol..

[62] Ivanov I.V., Bondarenko R.A. (2015). Individual Approach to the Use of Medications to Normalize Situational Anxiety under the Psycho-Emotional Stress. Aviakosm. Ekolog. Med..

[63] Mahon B.P., Hendon A.M., Driscoll J.M., Rankin G.M., Poulsen S.A., Supuran C.T., McKenna R. (2015). Saccharin: A lead compound for structure-based drug design of carbonic anhydrase IX inhibitors. Bioorg. Med. Chem..

[64] Kempf D.J., Sham H.L., Marsh K.C., Flentge C.A., Betebenner D., Green B.E., McDonald E., Vasavanonda S., Saldivar A., Wideburg N.E. (1998). Discovery of ritonavir, a potent inhibitor of HIV protease with high oral bioavailability and clinical efficacy. J. Med. Chem..

[65] Ikezoe T., Hisatake Y., Takeuchi T., Ohtsuki Y., Yang Y., Said J.W., Taguchi H., Koeffler H.P. (2004). HIV-1 protease inhibitor, ritonavir: A potent inhibitor of CYP3A4, enhanced the anticancer effects of docetaxel in androgen-independent prostate cancer cells in vitro and in vivo. Cancer Res..

[66] Gunthard H.F., Aberg J.A., Eron J.J., Hoy J.F., Telenti A., Benson C.A., Burger D.M., Cahn P., Gallant J.E., Glesby M.J. (2014). Antiretroviral treatment of adult HIV infection: 2014 recommendations of the International Antiviral Society-USA Panel. JAMA.

[67] Schlessinger A., Geier E., Fan H., Irwin J.J., Shoichet B.K., Giacomini K.M., Sali A. (2011). Structure-based discovery of prescription drugs that interact with the norepinephrine transporter, NET. Proc. Natl Acad. Sci. USA.

[68] Kim S., Thiessen P.A., Cheng T., Yu B., Shoemaker B.A., Wang J., Bolton E.E., Wang Y., Bryant S.H. (2016). Literature information in PubChem: Associations between PubChem records and scientific articles. J. Cheminform..

[69] Carvalho L., Luque-Ortega J.R., Manzano J.I., Castanys S., Rivas L., Gamarro F. (2010). Tafenoquine, an antiplasmodial 8-aminoquinoline, targets leishmania respiratory complex III and induces apoptosis. Antimicrob. Agents Chemother..

[70] Graves P.R., Kwiek J.J., Fadden P., Ray R., Hardeman K., Coley A.M., Foley M., Haystead T.A. (2002). Discovery of novel targets of quinoline drugs in the human purine binding proteome. Mol. Pharmacol..

[71] Chu C.S., Bancone G., Nosten F., White N.J., Luzzatto L. (2018). Primaquine-induced haemolysis in females heterozygous for G6PD deficiency. Malar. J..

[72] Anden N.E., Rubenson A., Fuxe K., Hokfelt T. (1967). Evidence for dopamine receptor stimulation by apomorphine. J. Pharm. Pharmacol..

[73] Anden N.E., Strombom U. (1974). Adrenergic receptor blocking agents: Effects on central noradrenaline and dopamine receptors and on motor activity. Psychopharmacologia.

[74] Warrilow A.G., Parker J.E., Kelly D.E., Kelly S.L. (2013). Azole affinity of sterol 14alpha-demethylase (CYP51) enzymes from Candida albicans and Homo sapiens. Antimicrob. Agents Chemother..

[75] Thomson S., Rice C.A., Zhang T., Edrada-Ebel R., Henriquez F.L., Roberts C.W. (2017). Characterisation of sterol biosynthesis and validation of 14alpha-demethylase as a drug target in Acanthamoeba. Sci. Rep..

[76] Carrillo-Munoz A.J., Tur-Tur C., Giusiano G., Marcos-Arias C., Eraso E., Jauregizar N., Quindos G. (2013). Sertaconazole: An antifungal agent for the topical treatment of superficial candidiasis. Expert Rev. Anti Infect. Ther..

[77] Warrilow A.G., Hull C.M., Rolley N.J., Parker J.E., Nes W.D., Smith S.N., Kelly D.E., Kelly S.L. (2014). Clotrimazole as a potent agent for treating the oomycete fish pathogen Saprolegnia parasitica through inhibition of sterol 14alpha-demethylase (CYP51). Appl. Environ. Microbiol..

[78] Pace J.R., DeBerardinis A.M., Sail V., Tacheva-Grigorova S.K., Chan K.A., Tran R., Raccuia D.S., Wechsler-Reya R.J., Hadden M.K. (2016). Repurposing the Clinically Efficacious Antifungal Agent Itraconazole as an Anticancer Chemotherapeutic. J. Med. Chem..

[79] Odds F.C., Brown A.J., Gow N.A. (2003). Antifungal agents: Mechanisms of action. Trends Microbiol..

[80] Makepeace B.L., Tanya V.N. (2016). 25 Years of the Onchocerca ochengi Model. Trends Parasitol..

[81] Gardon J., Gardon-Wendel N., Demanga N., Kamgno J., Chippaux J.P., Boussinesq M. (1997). Serious reactions after mass treatment of onchocerciasis with ivermectin in an area endemic for Loa loa infection. Lancet.

[82] Drugs for Neglected Diseases Initiative Filaria: River blindness, Oxfendazole. https://dndi.org/research-development/portfolio/oxfendazole/.

[83] Drugs for Neglected Diseases Initiative Filaria: River Blindness, Emodepside. https://dndi.org/research-development/portfolio/emodepside/.

[84] Hubner M.P., Martin C., Specht S., Koschel M., Dubben B., Frohberger S.J., Ehrens A., Fendler M., Struever D., Mitre E. (2020). Oxfendazole mediates macrofilaricidal efficacy against the filarial nematode Litomosoides sigmodontis in vivo and inhibits Onchocerca spec. motility in vitro. PLoS Negl. Trop. Dis..

[85] Kashyap S.S., Verma S., Voronin D., Lustigman S., Kulke D., Robertson A.P., Martin R.J. (2019). Emodepside has sex-dependent immobilizing effects on adult Brugia malayi due to a differentially spliced binding pocket in the RCK1 region of the SLO-1 K channel. PLoS Pathog..

[86] Maertens J.A. (2004). History of the development of azole derivatives. Clin. Microbiol. Infect..

[87] Gaulton A., Hersey A., Nowotka M., Bento A.P., Chambers J., Mendez D., Mutowo P., Atkinson F., Bellis L.J., Cibrian-Uhalte E. (2017). The ChEMBL database in 2017. Nucleic Acids Res..

[88] Wishart D.S., Feunang Y.D., Guo A.C., Lo E.J., Marcu A., Grant J.R., Sajed T., Johnson D., Li C., Sayeeda Z. (2018). DrugBank 5.0: A major update to the DrugBank database for 2018. Nucleic Acids Res..

[89] Tyagi R., Elfawal M.A., Wildman S.A., Helander J., Bulman C.A., Sakanari J., Rosa B.A., Brindley P.J., Janetka J.W., Aroian R.V. (2019). Identification of small molecule enzyme inhibitors as broad-spectrum anthelmintics. Sci. Rep..

[90] Seidler J., McGovern S.L., Doman T.N., Shoichet B.K. (2003). Identification and prediction of promiscuous aggregating inhibitors among known drugs. J. Med. Chem..

[91] Choi S.R., Pradhan A., Hammond N.L., Chittiboyina A.G., Tekwani B.L., Avery M.A. (2007). Design, synthesis, and biological evaluation of Plasmodium falciparum lactate dehydrogenase inhibitors. J. Med. Chem..

[92] Mast N., Zheng W., Stout C.D., Pikuleva I.A. (2013). Antifungal Azoles: Structural Insights into Undesired Tight Binding to Cholesterol-Metabolizing CYP46A1. Mol. Pharmacol..

[93] Janes J., Young M.E., Chen E., Rogers N.H., Burgstaller-Muehlbacher S., Hughes L.D., Love M.S., Hull M.V., Kuhen K.L., Woods A.K. (2018). The ReFRAME library as a comprehensive drug repurposing library and its application to the treatment of cryptosporidiosis. Proc. Natl. Acad. Sci. USA.

[94] Eder J., Hommel U., Cumin F., Martoglio B., Gerhartz B. (2007). Aspartic proteases in drug discovery. Curr. Pharm. Des..

[95] Nguyen J.T., Hamada Y., Kimura T., Kiso Y. (2008). Design of potent aspartic protease inhibitors to treat various diseases. Arch. Pharm. (Weinheim).

[96] WHO (2019). The Selection and Use of Essential Medicines: Report of the WHO Expert Committee on Selection and Use of Essential Medicines, 2019 (Including the 21st WHO Model List of Essential Medicines and the 7th WHO Model List of Essential Medicines for Children).

[97] Tort J., Brindley P.J., Knox D., Wolfe K.H., Dalton J.P. (1999). Proteinases and associated genes of parasitic helminths. Adv. Parasitol..

[98] Xu J., Liu R.D., Bai S.J., Hao H.N., Yue W.W., Xu Y.X.Y., Long S.R., Cui J., Wang Z.Q. (2020). Molecular characterization of a Trichinella spiralis aspartic protease and its facilitation role in larval invasion of host intestinal epithelial cells. PLoS Negl. Trop. Dis..

[99] Park J.N., Park S.K., Cho M.K., Park M.K., Kang S.A., Kim D.H., Yu H.S. (2012). Molecular characterization of 45 kDa aspartic protease of Trichinella spiralis. Vet. Parasitol..

[100] Williamson A.L., Brindley P.J., Abbenante G., Datu B.J., Prociv P., Berry C., Girdwood K., Pritchard D.I., Fairlie D.P., Hotez P.J. (2003). Hookworm aspartic protease, Na-APR-2, cleaves human hemoglobin and serum proteins in a host-specific fashion. J. Infect. Dis..

[101] Jolodar A., Fischer P., Buttner D.W., Miller D.J., Schmetz C., Brattig N.W. (2004). Onchocerca volvulus: Expression and immunolocalization of a nematode cathepsin D-like lysosomal aspartic protease. Exp. Parasitol..

[102] Hamada Y., Kiso Y. (2016). New directions for protease inhibitors directed drug discovery. Biopolymers.

[103] Drag M., Salvesen G.S. (2010). Emerging principles in protease-based drug discovery. Nat. Rev. Drug Discov..

[104] Monika S., Malgorzata B., Zbigniew O. (2017). Contribution of Aspartic Proteases in Candida Virulence. Protease Inhibitors against Candida Infections. Curr. Protein Pept. Sci..

[105] Castilho V.V.S., Goncalves K.C.S., Rebello K.M., Baptista L.P.R., Sangenito L.S., Santos H.L.C., Branquinha M.H., Santos A.L.S., Menna-Barreto R.F.S., Guimaraes A.C. (2018). Docking simulation between HIV peptidase inhibitors and Trypanosoma cruzi aspartyl peptidase. BMC Res. Notes.

[106] Rawlings N.D., Barrett A.J., Finn R. (2016). Twenty years of the MEROPS database of proteolytic enzymes, their substrates and inhibitors. Nucleic Acids Res..

[107] Benes P., Vetvicka V., Fusek M. (2008). Cathepsin D-many functions of one aspartic protease. Crit. Rev. Oncol. Hematol..

[108] Sarkis G.J., Kurpiewski M.R., Ashcom J.D., Jen-Jacobson L., Jacobson L.A. (1988). Proteases of the nematode Caenorhabditis elegans. Arch. Biochem. Biophys..

[109] Lv Z., Chu Y., Wang Y. (2015). HIV protease inhibitors: A review of molecular selectivity and toxicity. HIV AIDS (Auckl.).

[110] Cotton J.A., Bennuru S., Grote A., Harsha B., Tracey A., Beech R., Doyle S.R., Dunn M., Hotopp J.C., Holroyd N. (2016). The genome of *Onchocerca volvulus*, agent of river blindness. Nat. Microbiol..

[111] Rigobello M.P., Scutari G., Boscolo R., Bindoli A. (2002). Induction of mitochondrial permeability transition by auranofin, a gold(I)-phosphine derivative. Br. J. Pharmacol..

[112] Njouendou A.J., Ritter M., Ndongmo W.P.C., Kien C.A., Narcisse G.T.V., Fombad F.F., Tayong D.B., Pfarr K., Layland L.E., Hoerauf A. (2017). Successful long-term maintenance of Mansonella perstans in an in vitro culture system. Parasites Vectors.

[113] Verma M., Pathak M., Shahab M., Singh K., Mitra K., Misra-Bhattacharya S. (2014). Moxidectin causes adult worm mortality of human lymphatic filarial parasite Brugia malayi in rodent models. Folia Parasitol. (Praha).

[114] Verma S., Kashyap S.S., Robertson A.P., Martin R.J. (2017). Functional genomics in Brugia malayi reveal diverse muscle nAChRs and differences between cholinergic anthelmintics. Proc. Natl. Acad. Sci. USA.

[115] Partridge F.A., Forman R., Bataille C.J.R., Wynne G.M., Nick M., Russell A.J., Else K.J., Sattelle D.B. (2020). Anthelmintic drug discovery: Target identification, screening methods and the role of open science. Beilstein J. Org. Chem..

[116] Marcellino C. GitHub Repository. https://github.com/chrismarcellino/wormassay..

[117] Bulman C.A., Bidlow C.M., Lustigman S., Cho-Ngwa F., Williams D., Rascon A.A., Tricoche N., Samje M., Bell A., Suzuki B. (2015). Repurposing auranofin as a lead candidate for treatment of lymphatic filariasis and onchocerciasis. PLoS Negl. Trop. Dis..

[118] Bickerton G.R., Paolini G.V., Besnard J., Muresan S., Hopkins A.L. (2012). Quantifying the chemical beauty of drugs. Nat. Chem..

[119] Harris T.W., Arnaboldi V., Cain S., Chan J., Chen W.J., Cho J., Davis P., Gao S., Grove C.A., Kishore R. (2020). WormBase: A modern Model Organism Information Resource. Nucleic Acids Res..

[120] Emms D.M., Kelly S. (2019). OrthoFinder: Phylogenetic orthology inference for comparative genomics. Genome Biol..

[121] Katoh K., Standley D.M. (2013). MAFFT multiple sequence alignment software version 7: Improvements in performance and usability. Mol. Biol. Evol..

[122] Waterhouse A.M., Procter J.B., Martin D.M., Clamp M., Barton G.J. (2009). Jalview Version 2—A multiple sequence alignment editor and analysis workbench. Bioinformatics.

[123] Guindon S., Dufayard J.F., Lefort V., Anisimova M., Hordijk W., Gascuel O. (2010). New algorithms and methods to estimate maximum-likelihood phylogenies: Assessing the performance of PhyML 3.0. Syst. Biol..

[124] Cao Y., Charisi A., Cheng L.C., Jiang T., Girke T. (2008). ChemmineR: A compound mining framework for R. Bioinformatics.

